# Proteomic analysis of bovine mammary epithelial cells after in vitro incubation with *S. agalactiae*: potential biomarkers

**DOI:** 10.1186/s13567-020-00808-7

**Published:** 2020-08-03

**Authors:** Jinjin Tong, Mingwei Sun, Hua Zhang, Delian Yang, Yonghong Zhang, Benhai Xiong, Linshu Jiang

**Affiliations:** 1 Beijing Key Laboratory for Dairy Cow Nutrition, Beijing University of Agriculture, Beijing, 102206 People’s Republic of China; 2 State Key Laboratory of Animal Nutrition, Institute of Animal Science, Chinese Academy of Agricultural Sciences, Beijing, 100193 People’s Republic of China

**Keywords:** proteomic analysis, bovine mammary epithelial cells, *Streptococcus agalactiae*, potential biomarkers

## Abstract

*Streptococcus agalactiae* is one of the causative agents of subclinical mastitis, a common disease of dairy cows that causes great economic losses in the industry worldwide. It is thought that pathology is mainly due to inflammatory damage of bovine mammary epithelial cells (bMECs); however, the mechanism by which *S. agalactiae* damages the bMECs is not clear. The aim of this study was to evaluate the inflammatory effects of *S. agalactiae* on bMECs and the resulting changes in protein profiles. The bMECs were incubated with *S. agalactiae* for different times and assayed for cell viability by MTT assay, apoptosis by annexin V and propidium iodide dual staining, and morphological and ultrastructural changes by scanning and transmission electron microscopy. Quantitative real-time PCR was used to determine the effect of *S. agalactiae* on expression of mRNA of inflammatory factors in bMECs and protein levels were quantitated by liquid chromatography/mass spectrometry. Exposure to *S. agalactiae* significantly decreased the cell viability and triggered apoptosis, as well as up-regulating TNF-α, IL-1β and IL-6 mRNA, and inhibiting IL-8 expression. *S. agalactiae* also induced morphological and ultrastructural changes. Furthermore, we identified 325 up-regulated and 704 down-regulated proteins in the treated vs control group. All significant differentially expressed proteins (DSEPs) were classified into three major areas by function: biological processes, cellular components and molecular functions. These differentially expressed proteins included enzymes and proteins associated with various metabolic processes and cellular immunity. Pathway enrichment analysis showed that eight down-regulated signaling pathways were significantly enriched. Exposure to even subclinical levels of *S. agalactiae* can lead to inflammation and bMEC damage. Our data suggest some possible molecular mechanisms for the harmful effects of subclinical mastitis in dairy cows.

## Introduction

Mastitis is the most common disease in dairy cows. It has high incidence and prevalence within dairy herds ranging from 20 to 60% and causing more than $350 billion in losses in the dairy industry worldwide [1, 2]. Mastitis is an inflammation of the mammary glands in the udders of dairy cows that is mainly caused by infection with pathogenic microorganisms such as *Streptococcus agalactiae*, *Escherichia coli* and *Staphylococcus aureus*. Mastitis infections can be either clinical with obvious swelling and discharge, or subclinical with no overt signs. Subclinical mastitis (SCM) is the most economically detrimental of the two types because it is so common, often goes undiagnosed, and can cause an udder quarter with SCM to lose an average of 17.2% of its milk production [3].

Since inflammation of the mammary tissues is the primary cause of damage, it is necessary to examine the immune response to *S. agalactiae* infection to determine ways to mitigate it. The innate and cellular immune reactions related to mastitis are complex and involve the panoply of immune cells, the bMECs of the mammary gland tissue and the endothelial cells. The bMECs comprise 70% of the total udder cells and act as the dominant sentinel of the parenchyma as it mounts the first cytokine alert to bacterial invasion [4]. Once the pathogens penetrate the physical barrier of the teat canal, the host innate immune system detects the bacteria through pattern-recognition receptors (PRRs), particularly via the toll-like receptors (TLRs) [5]. Binding of microbial components to TLRs activates TLR signaling pathways that trigger several intracellular signal transduction cascades resulting in the production of pro-inflammatory chemokines such as interleukin-8 (IL-8), and cytokines like tumor necrosis factor-alpha (TNF-α), IL-6, and IL-1β leading to inflammation and eventually elimination of the pathogens by leukocytes [6].

The aim of this study was to identify the infective interactions of *S. agalactiae* with bMECs in vitro on the transcriptional and translational level by means of real-time polymerase chain reaction (PCR) and quantitative proteomics using tandem mass tag (TMT)-labeled LC–MS/MS and to use these protein profiles to identify potential biomarkers of the inflammatory effects and change in cell function.

## Materials and methods

### Mammalian cell and bacterial culture

The primary bovine mammary epithelial cells (bMECs) were kindly provided by Northeast Agricultural University and established as a cell line as described by Chen et al. [7].The bMECs were cultured in Dulbecco’s modified Eagle’s medium/F-12 (DMEM/F-12, Gibco) supplemented with 10% heat-inactivated fetal bovine serum (FBS, Gibco), 100 U/mL penicillin and 100 μg/mL streptomycin at 37 °C in a humidified atmosphere of 5% CO_2_/95% air. The medium was changed every two days. The *S. agalactiae* (CVCC 3940) strain used in this study was bought from the China Institute of Veterinary Drug Control and has the capacity to infect bMECs. The *S. agalactiae* were grown in brain–heart infusion broth (Oxoid, UK) at 37 °C for 24 h and the number of CFUs was determined by standard dilution and colony counting on tryptic soy agar plates.

### Cell viability assay

Cytotoxic effects on cell viability were measured by the MTT [3-(4, 5-dimethylthiazol-2-yl)-2, 5-diphenyltetrazolium] assay as previously described (Beyotime, Shanghai, China). The bMECs were inoculated into 96-well plates (3599, Costar) at 1 × 10^3^ cells/well and infected with *S. agalactiae* at a multiplicity of infection (MOI of 50:1 for 1, 2, 4, 6, 8 and 10 h) at 37 °C. *S. agalactiae* suspensions in DMEM/F12 with 4% FBS and non-infected cells incubated at the same time were used as controls.

The proliferation of bMECs was determined with the MTT cell proliferation and cytotoxicity assay kit (Beyotime, Shanghai, China). After incubation, the supernatants were removed and cells were gently washed three times with phosphate buffer saline (PBS, pH 7.4) to remove non-adherent bacteria. Then, 10 μL of MTT solution (5 mg/mL) was added to each well and plates were incubated for 4 h at 37 °C. After incubation, 100 μL of formazan solubilizer was added to each well and incubation was continued in the cell culture incubator until all of the formazan was dissolved. The absorbance of the plate was read at 570 nm in a microplate reader (Thermo Multiscan FC, Shanghai). All experiments were performed in triplicate.

### Cell apoptosis assay

The annexin V-FITC/PI apoptosis detection kit (Bio-Friend, Beijing, China) was used to detect programmed cell death according to the manufacturer’s instructions. *S. agalactiae* (MOI 50:1) were incubated with bMECs for 2, 6 and 12 h at 37 °C and cells without *S. agalactiae* treatment were used as control. The harvested cells were washed twice with cold PBS and resuspended in 250 μL binding buffer at a concentration of 1 × 10^6^ cells/mL. Aliquots of 100 μL of cell suspension were transferred into 5 mL flow tubes and stained with 5 μL annexin V-FITC and 5 μL propidium iodide. After incubation for 15 min at room temperature, 400 μL of PBS was added to the tubes and cell populations were analyzed within 1 h by flow cytometry (FACS-ACEA, China).

### Cell morphology and ultrastructural analysis

The bMEC morphology was visualized by scanning electron microscopy (SEM). The cells were grown on cover slips in 6-well dishes (5 × 10^5^ cells/well) and then incubated with *S. agalactiae* (MOI 50:1) for 2, 4, 6 and 8 h, and uninfected cells were used as control. The bMECs adhered on coverslips were washed three times with cold PBS and fixed with 2.5% glutaraldehyde at 4 °C overnight. Fixed cells were dehydrated in a graded series of 30, 50, 70, 80, 90, and 100% ethanol) for 15 min at room temperature and ethanol was displaced three times with t-butanol for 10 min each time. After lyophilization and gold coating, cells were viewed on a scanning electron microscope (JEOL JSM-6700F, Japan).

Cellular ultrastructure was analyzed by transmission electron microscopy (TEM). For TEM, cells were cultured in 6-well dishes and incubated with *S. agalactiae* for 6 h, and the pretreatment of the cells before dehydration was similar to that for SEM. After dehydration by graded ethanol and acetone (three times, 10 min each), cells were embedded in acetone-epoxy resin (2:1; 1:2) for 3–4 h and then overnight at room temperature, and finally embedded in pure epoxy resin for 2–3 h at 37 °C. After the resin polymerized, ultrathin sections were cut with a microtome (Leica EM UC7, Germany), stained with 3% uranium acetate-lead citrate, and viewed by TEM (Hitachi HT7700, Japan).

### RNA extraction and RT-PCR

The bMECs were incubated with *S. agalactiae* (MOI 50:1) for 1, 2, 4, 6, 8 h and non-infected cells were used as control. Total RNA was extracted using the Total RNA Kit I (Omega, Guangzhou, China) according to the manufacturer’s protocol. The absorbance values at 260 and 280 nm were read to assess RNA concentration and purity in the samples, and RNA integrity was assessed by electrophoresis on 2% agarose gels (m/v). The RNA (2 μg) was reverse transcribed into cDNA with the PrimeScriptTM RT reagent Kit (TaKaRa, Japan). Quantitative real-time PCR (qRT-PCR) reactions were performed using SYBR green Premix Ex TaqII (Tli RNaseH Plus) (TaKaRa, Japan) on a Roche LightCycler 96 (Basel, Switzerland) in a total volume of 20 μL using 96-well plates. The primers used in this experiment are shown in Table 1. All target mRNAs were normalized to β-actin mRNA level. The relative expression of the target genes was quantified using the 2^−ΔΔCt^ method.

**Table 1:** **Oligonucleotides used in this study**

Primer	Sequence, 5′-3′	Size (bp)	Source
β-actin forward primer	TCACCAACTGGGACGACA	206	Fu et al. [37]
β-actin reverse primer	GCATACAGGGACAGCACA		
TNF-α forward primer	TCTTCTCAAGCCTCAAGTAACAAGC	104	Lahouassa et al. [23]
TNF-α reverse primer	CCATGAGGGCATTGGCATAC		
IL-1β forward primer	CTCTCACAGGAAATGAACCGAG	152	Lahouassa et al. [23]
IL-1β reverse primer	GCTGCAGGGTGGGCGTATCACC		
IL-6 forward primer	ATGCTTCCAATCTGGGTTC	269	Fu et al. [37]
IL-6 reverse primer	TGAGGATAATCTTTGCGTTC		
IL-8 forward primer	ACACATTCCACACCTTTCCA	124	Fu et al. [37]
IL-8 reverse primer	GGTTTAGGCAGACCTCGTTT		

### Protein extraction and proteomic analysis

After 6 h incubation, cells were harvested by trypsin digestion, and sonicated three times on ice using a high-intensity ultrasonic processor (Scientz) in lysis buffer containing 8 M urea, and 1% protease inhibitor cocktail. The supernatant was collected and the protein concentration was determined with a BCA kit according to the manufacturer’s instructions. The protein lysates were reduced with 5 mM dithiothreitol for 30 min at 56 °C and alkylated with 11 mM iodoacetamide for 15 min at room temperature in darkness. The lysates were then diluted by adding 100 mM triethylammonium bicarbonate (TEAB) with a final urea concentration of less than 2 M. Finally, trypsin was added at 1:50 trypsin-to-protein mass ratio for the first digestion overnight at 37 °C and 1:100 trypsin-to-protein mass ratio for a second digestion of 4 h. After trypsin digestion, the samples were desalted on a Strata X C18 SPE column (Phenomenex) and vacuum-dried. The peptide digest was reconstituted in 0.5 M TEAB and processed according to the manufacturer’s protocol for TMT labeling.

### HPLC fractionation and LC–MS/MS analysis

The tryptic peptides were separated into fractions by high-pH reverse-phase HPLC on an Agilent 300Extend-C18 column (5 μm particle size, 4.6 mm ID, 250 mm length) using a chromatograph (Thermo EASY-nLC 1000 Nano HPLC, USA) and dissolved in 0.1% formic acid (solvent A). Samples were directly loaded onto a homemade reversed-phase analytical liquid chromatography column (15-cm length, 75 μm i.d.) connected to an EASY-nLC 1000 UPLC system. The gradient consisted of an increase from 6 to 23% solvent B (0.1% formic acid in 98% acetonitrile) over 26 min, 23% to 35% in 8 min and increasing to 80% in 3 min, then holding at 80% for the last 3 min, all at a constant flow rate of 400 nL/min. Then the peptides were subjected to a nanospray ionization source followed by tandem mass spectrometry (MS/MS) on a Q ExactiveTM Plus (Thermo) coupled online to the UPLC.

The applied electrospray voltage was 2.0 kV. The m/z scan range was 350 to 1800 for full scan, and intact peptides were detected by the Orbitra*P* at a resolution of 70,000. Peptides were then selected for MS/MS using an NCE setting of 28 and the fragments were detected in the Orbitra*P* at a resolution of 17,500. The data-dependent procedure alternated between one MS scan followed by 20 MS/MS scans with a 15.0 s dynamic exclusion. The automatic gain control (AGC) was set at 5E4 and the fixed first mass was set at 100 m/z.

### Proteomic data analysis

The resulting MS/MS data were processed using the Maxquant search engine (v.1.5.2.8). The tandem mass spectra were searched against the UniProt *Bos taurus* database concatenated with a reverse decoy database. Gene Ontology (GO) software was used to analyze enrichment for signaling pathway proteins and for the putative protein functionality present in and between each sample (http://www.ebi.ac.uk/GOA/). The Kyoto Encyclopedia of Genes and Genomes (KEGG) Pathway Annotation was used to determine the number of pathways detected in each sample and the number of proteins of each pathway represented in each sample (http://www.genome.jp/kegg/).

### Statistical analysis

All experiments were performed independently at least three times and the results are presented as mean ± standard error of the mean (SEM). Statistical analysis was performed using Student’s *t* test. Statistical significance was defined as *p *< 0.05.

## Results

### Viability and apoptosis of bMECs after *S. agalactiae* incubation

**Figure 1:**
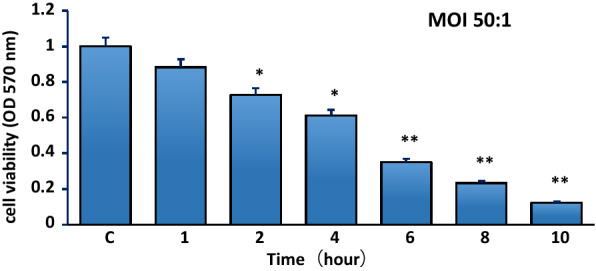
**Cytotoxicity of*S. agalactiae*to bMECs.** Cell viability was determined by MTT assay. Values are presented as mean ± SEM. *p < 0.05, **p < 0.01 as compared with the control group.

Incubation with *S. agalactiae* caused a significant decrease in the viability of bMECs from 27.1% loss at 2 h (*p *< 0.05) to 87.9% after 12 h exposure (*p* < 0.01) compared to control cells in the absence of bacteria (Figure 1).

**Figure 2:**
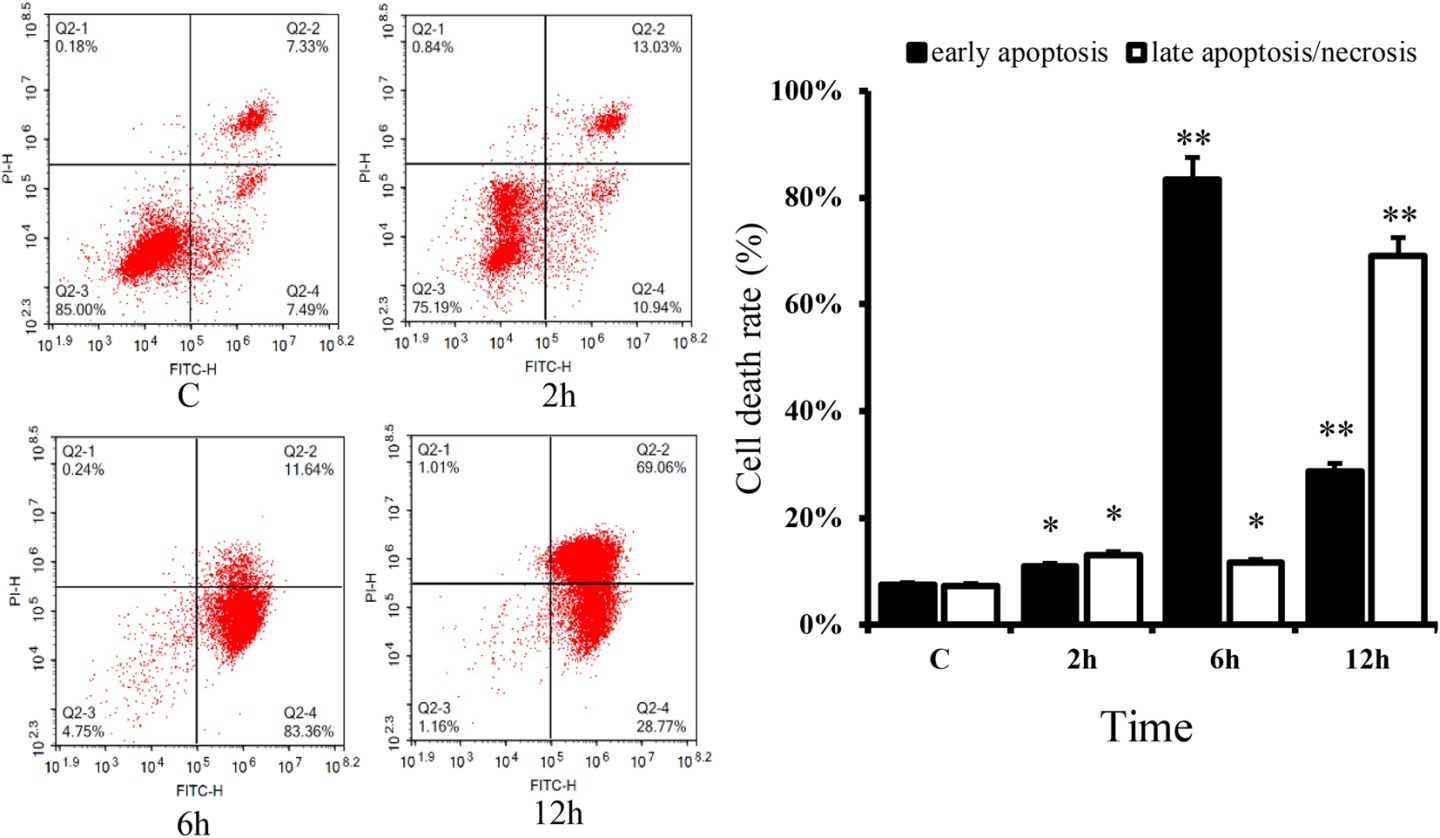
**Apoptosis and necrosis of bMECs analyzed by flow cytometry with annexin V/propidium iodide (PI) dual staining. A** Two dimensional scatter plots of FITC annexin V vs. PI from flow cytometry. Cells staining negative for FITC annexin V and PI in the lower left quadrant are live cells. Cells staining positive for FITC annexin V and negative for PI in the lower right quadrant represent early apoptosis. Cells staining positive for both FITC annexin V and PI in the upper right quadrant are the late apoptotic/necrotic cells. **B** Percentage of early apoptotic cells and late apoptotic/necrotic cells. Data are presented as mean ± SD of three independent experiments. *p < 0.05, **p < 0.01 as compared with the control group.

The induction of apoptosis in bMECs was slightly increased after 2 h with *S. agalactiae* (*p *< 0.05) compared to uninfected controls, but from 6 to 12 h exposure, the number of apoptotic/necrotic cells in early apoptosis increased to 83.36% (*p *< 0.01). The apoptosis of BMECs induced by *S. agalactiae*, especially early apoptosis, was significantly increased in a time-dependent manner (Figure 2).

### Morphological and ultrastructural analysis of bMECs

**Figure 3:**
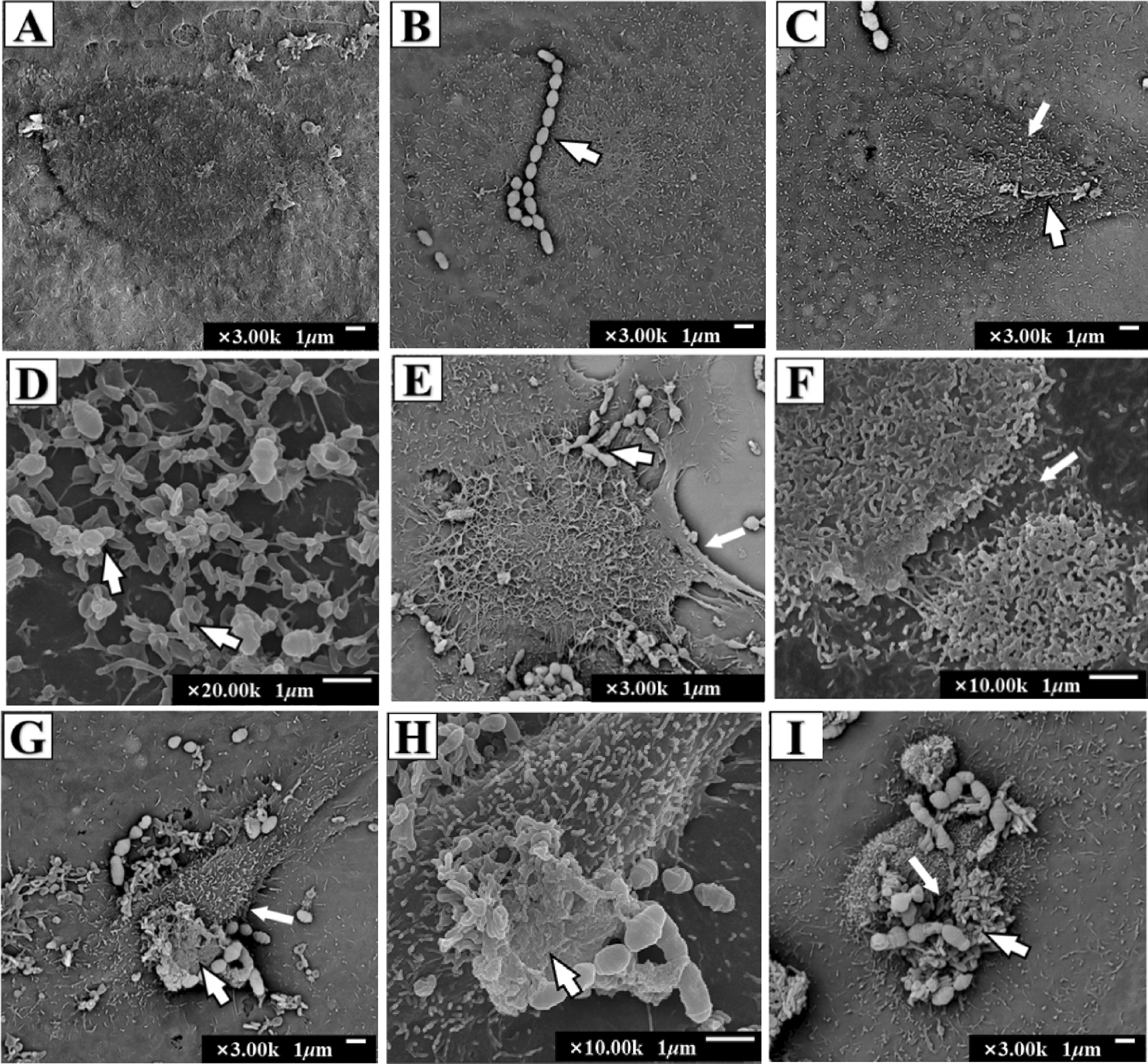
**Scanning electron photomicrographs showing interaction of bMECs infected with*S. agalactiae***. **A** Untreated bMECs; **B***S. agalactiae* (arrow); **C** cell shrinkage and slight bulging at 2 h. **D** Microvilli trapping *S. agalactiae* at 2 h (arrows). **E** A cell with folded edge (thick arrow) and microvilli extending and wrapping *S. agalactiae* (thin arrow) at 4 h. **F** Cell fracture at 4 h; **G** cells with obvious shrinkage, dysmorphosis, desquamation and microvilli disappearance (thin arrow) at 6 h; **H***S. agalactiae* secrete substances that adhere to the cell surface at 6 h (arrow); and **I** cell membrane breakage at 8 h.

Scanning electron microscopy (SEM) was used to explore the interaction between bMECs and *S. agalactiae* and detect any overt cell changes. Control bMECs without bacterial infection showed no morphological changes at the end of the incubation period (Figure 3A). *S. agalactiae* appeared as single or multiple chains (Figure 3B). At 2 h infection, some bMECs showed slight shrinkage (Figure 3C) and *S. agalactiae* adherence (Figure 3D). After 4 and 6 h infection, there was obvious cellular shrinkage, dysmorphosis, desquamation and cell adhesion edge deterioration; even some cell disruption (Figure 3E–H). After 4 h infection, cell microvilli elongated and wrapped *S. agalactiae* (Figure 3E), but most of cell microvilli disappear after 6 h infection and *S. agalactiae* adhere to the cell surface (Figure 3G, H). Finally, most cells were breaking up by 8 h infection. It appears that *S. agalactiae* adhere to the cell surface and secrete toxic substances that cause cell damage.

**Figure 4:**
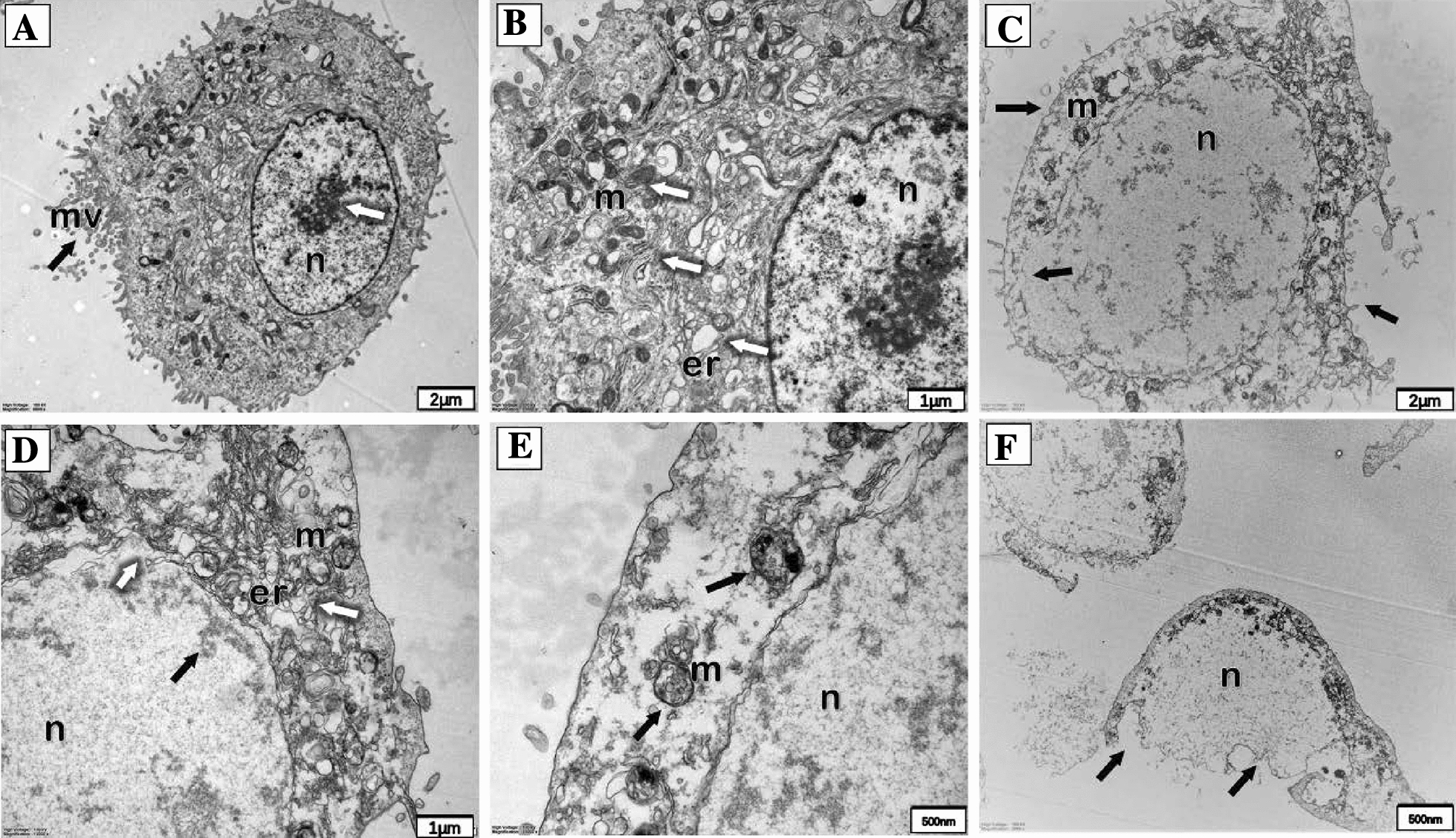
**Transmission electron photomicrographs showing pathological ultrastructural changes in bMECs infected with*S. agalactiae***. **A**, **B** Non-infected bMECs are rich in microvilli and have abundant organelles in cytoplasm, especially mitochondria; **C** after 6 h infection, bMECs show deformation, loss of microvilli, nuclear swelling and rupture (black arrows); **D** Organelle disruption and swollen endoplasmic reticulum (white arrows), chromatin spread to the nuclear membrane edge (black arrow). **E** Mitochondria with decreased electron density, cristae degeneration and accumulation of dense granules. **F** Some cells show disruption and loss of organelles. *n* nucleus, *m* mitochondrion, *mv* microvillus, *er* endoplasmic reticulum.

Transmission electron microscopy (TEM) was used to visualize the ultrastructural changes in bMECs with *S. agalactiae* infection. In the control group, cells have a complete structure with clear cell membrane rich in microvilli, distinct cytoplasmic and nuclear areas and abundant mitochondria (Figures 4A, B). After 6 h infection with *S. agalactiae*, the ultrastructure was markedly changed, including organelle disorder, loss of microvilli and also expansion of the perinuclear space (Figure 4C). From (Figures 4D–F), we can see swollen endoplasmic reticulum (d), cristae degeneration and accumulation of dense granules in mitochondria (e), chromatin fragmentation and spreading to the nuclear membrane edge (c), and rupturing of the cell membrane (f).

### Expression of pro-inflammatory cytokines by bMECs

**Figure 5:**
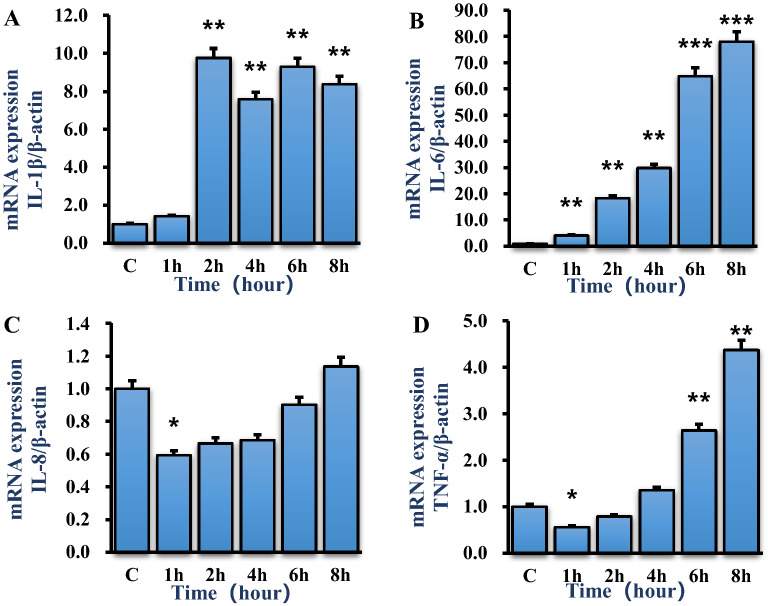
***S. agalactiae*infection increases mRNA expression of IL-1β, IL-6, IL-8, TNF-α. A** Relative mRNA expression of IL-1β. **B** Relative mRNA expression of IL-6. **C** Relative mRNA expression of TNF-α. **D** Relative mRNA expression of IL-8. Results are presented as mean ± SEM of three independent experiments. *p < 0.05, **p < 0.01, ***p < 0.001 as compared with the control group.

The mRNA expression of TNF-α, IL-6, IL-8 and IL-1β in bMECs induced by *S. agalactiae* was determined by RT-PCR. Results showed that the expression of IL-1β was significantly upregulated after 2 h infection (*p* < 0.01) (Figure 5A). IL-6 mRNA expression was markedly upregulated by *S. agalactiae* in a time-dependent manner after 1 h post-infection (*p *< 0.01) (Figure 5B). Strikingly, no difference was observed in IL-8 mRNA expression except that it was significantly downregulated at 1 h (*p* < 0.05) (Figure 5C). Compared with control, the TNF-α expression was significantly downregulated at 1 h (*p* < 0.05) but was much higher after 6 and 8 h of *S. agalactiae* infection (*p* < 0.01) (Figure 5D).

### Protein identification and quantification using TMT-LC–MS/MS

Two experimental groups containing six different samples were analyzed using TMT labeling to quantitate protein levels. A total of 5239 proteins was identified, of which 4601 contained quantitative information. A 1.5-fold threshold and t-test (*p* < 0.05) were used to establish valid protein changes. Among the proteins identified from bMECss incubated with *S. agalactiae* and quantified, 325 were up-regulated and 704 were down-regulated.

**Figure 6:**
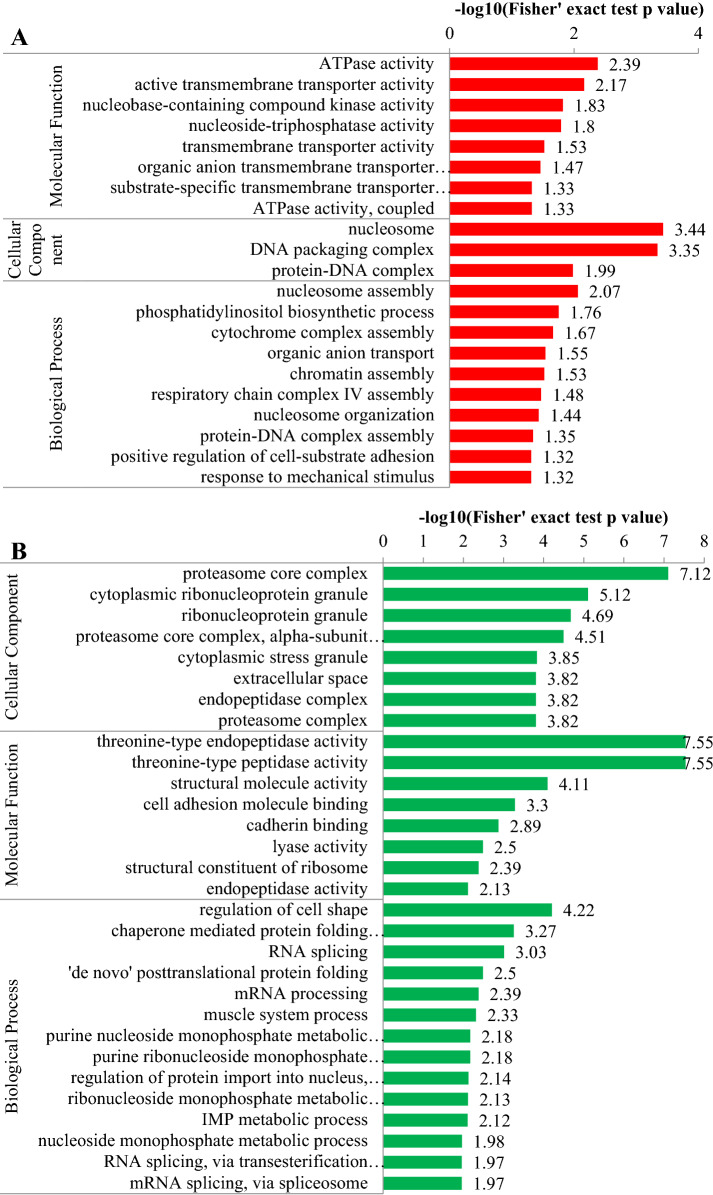
**GO enrichment of up-regulated proteins (A) and down-regulated proteins (B) (T/C)**.

We performed statistics on the distribution of quantified proteins using GO secondary annotations classification, including three major classes: biological processes (BP), cellular components (CC), and molecular functions [8] (Tables 2 and 3). By using GO enrichment analysis of the functions of the differentially expressed proteins, we found among the up-regulated proteins that the top eight GO terms for MF (*p* < 0.01) included ATPase, active transmembrane transporters, nucleobase-containing compound kinases, nucleoside-triphosphatases, etc. The top three GO terms for CC (*p *< 0.01) were nucleosomes, DNA packaging complexes, and protein-DNA complexes. There were ten GO terms for BP involving nucleosome assembly, phosphatidylinositol biosynthesis, cytochrome complex assembly, organic anion transport, etc. (Figure 6A). Among the down-regulated proteins, the top eight GO terms for CC were proteasome core complex, cytoplasmic ribonucleoprotein granules, etc. The top eight GO terms for MF include threonine-type endopeptidase activity, threonine-type peptidase activity, etc.; the top 14 GO terms for BP are regulation of cell shape, chaperone-mediated protein folding independent of cofactor, etc. (Figure 6B).

**Table 2:** **Distribution of up-regulated proteins in GO secondary annotations (T/C)**

GO terms level 1	GO terms level 2	No. of proteins
Biological process	Cellular process	228
	Metabolic process	160
	Single-organism process	153
	Biological regulation	141
	Cellular component organization or biogenesis	99
	Response to stimulus	85
	Localization	79
	Multicellular organismal process	55
	Developmental process	54
	Signaling	49
	Immune system process	17
	Multi-organism process	15
	Other	31
Cellular component	Cell	266
	Organelle	219
	Membrane	141
	Macromolecular complex	108
	Membrane-enclosed lumen	87
	Extracellular region	48
	Cell junction	9
	Other	10
Molecular function	Binding	221
	Catalytic activity	119
	Molecular function regulator	25
	Transporter activity	21
	Structural molecule activity	14
	Molecular transducer activity	8
	Signal transducer activity	7
	Transcription factor activity, protein binding	7
	Other	6

**Table 3:** **Distribution of down-regulated proteins in GO secondary annotations (T/C)**

GO terms level 1	GO terms level 2	No. of proteins
Biological process	Cellular process	463
	Single-organism process	318
	Biological regulation	318
	Metabolic process	314
	Cellular component organization or biogenesis	191
	Response to stimulus	152
	Localization	131
	Multicellular organismal process	123
	Developmental process	121
	Signaling	77
	Multi-organism process	35
	Immune system process	34
	Biological adhesion	27
	Other	58
Cellular component	Cell	588
	Organelle	521
	Macromolecular complex	236
	Membrane	220
	Membrane-enclosed lumen	198
	Extracellular region	193
	Cell junction	65
	Supramolecular complex	45
	Other	17
Molecular function	Binding	506
	Catalytic activity	180
	Structural molecule activity	55
	Molecular function regulator	46
	Nucleic acid binding transcription factor activity	17
	Transporter activity	17
	Transcription factor activity, protein binding	16
	Signal transducer activity	10
	Other	27

**Figure 7:**
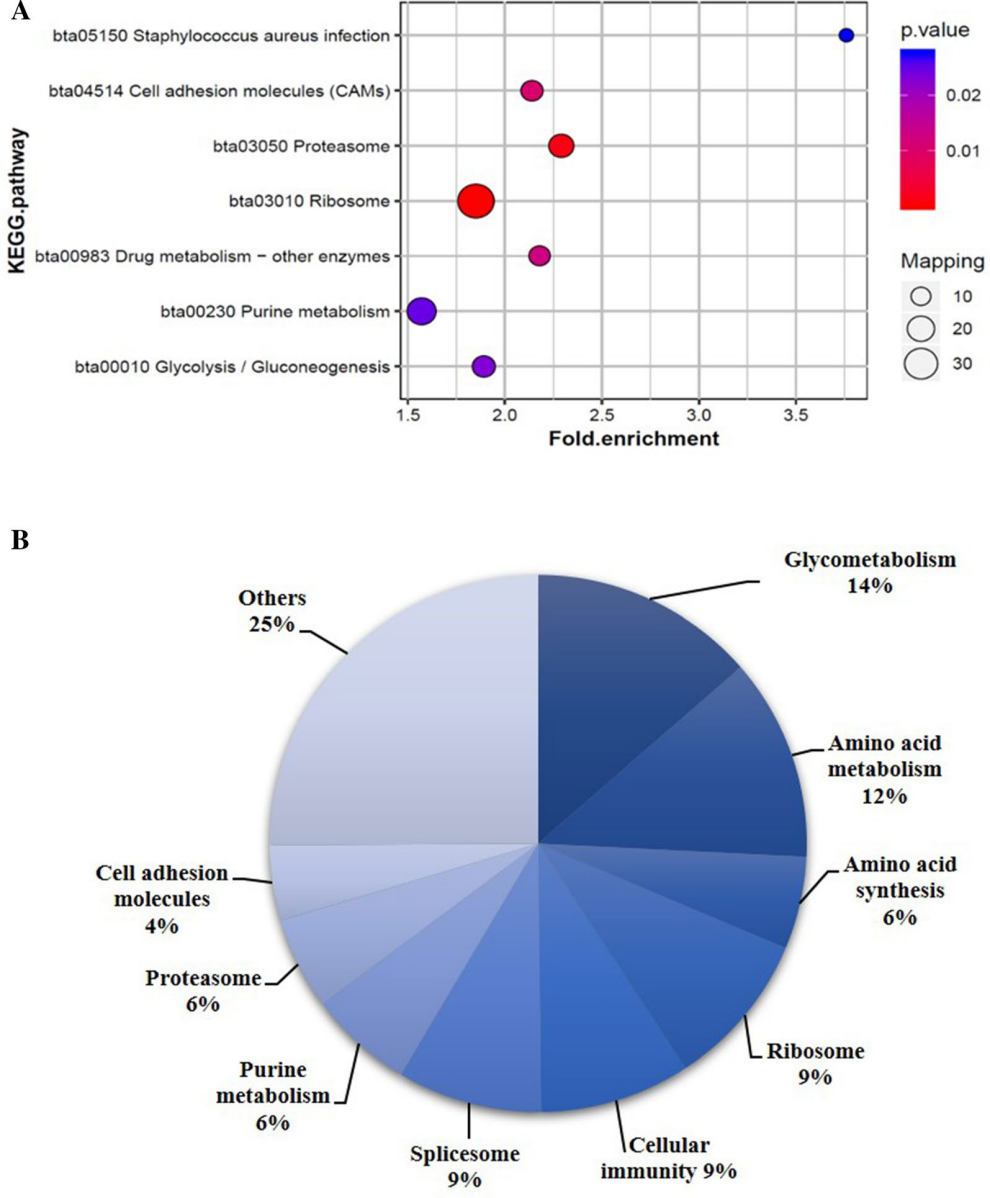
**KEGG pathway enrichment**

Pathway enrichment analysis showed that eight down-regulated signaling pathways were significantly enriched: proteasome, cell adhesion molecules (CAMs), ribosome, glycolysis/gluconeogenesis, spliceosome, *Staphylococcus aureus* infection, pentose phosphate pathway and purine metabolism (Figure 7A). In addition, among the down-regulated proteins, 287 participated in 42 specific pathways, including glycometabolism (14%), amino acid synthesis and metabolism (18%), ribosomes (9%), cellular immunity (9%), spliceosomes (9%), purine metabolism (6%), proteasomes (6%) and cell adhesion molecules (4%) (Figure 7B). In addition, among the top ten differentially expressed up- or down-regulated proteins there were several uncharacterized ones (Tables 4 and 5). Some proteins with special functions mentioned in some studies like dimethylarginine dimethylaminohydrolase 1 (DDAH1), peroxiredoxin-6 (PRDX6), epithelial cell adhesion molecule (EPCAM), were down-regulated.

**Table 4:** **Top 10 differentially up-regulated proteins**

Gene name	Protein description	T/C ratio	T/C *p* value
C3orf58	Uncharacterized protein	95.822	0.0090228
CLYBL	Uncharacterized protein	66.21	0.0173614
CFAP46	Uncharacterized protein	34.48	0.0038223
MATN4	Uncharacterized protein	33.407	6.7991E−08
RERE	Uncharacterized protein	32.414	0.00099539
PDE11A	Phosphodiesterase	30.873	0.000019091
SEL1L3	Uncharacterized protein	27.238	6.0201E−08
KMT5C	Uncharacterized protein	23.274	0.0026386
DHX35	Uncharacterized protein	23.18	2.3707E−06
SENP6	Uncharacterized protein	20.006	1.1863E−06

**Table 5:** **Top 10 differentially down-regulated proteins**

Gene name	Protein description	T/C ratio	T/C *p* value
ZFAND2B	Uncharacterized protein	0.05	0.000020886
H2B	Histoneh2B	0.075	0.000020316
TNRC6B	Uncharacterized protein	0.083	3.6866E−08
WIPF2	Uncharacterized protein	0.087	0.00020302
FAM107B	Protein FAM107B	0.101	8.7112E−08
HMGN2	Non-histone chromosomal proteinhMG-17	0.117	1.4851E−06
BTF3	Transcription factor BTF3	0.128	2.2885E−07
ENSA	Alpha-endosulfine	0.129	2.0782E−07
DAP	Death-associated protein 1	0.133	0.000096505
SRRM1	Uncharacterized protein	0.135	2.7367E−07

## Discussion

In this study, we hypothesized that the interaction of *S. agalactiae* with bMECs caused inflammatory effects and changes in cell function that were reflected in altered expression of proteins, some of which may be used as potential biomarkers for diagnosis of mastitis. This hypothesis was supported by the finding that *S. agalactiae* significantly affected the viability of bMECs in a time-dependent manner. *S. agalactiae* could adhere to the cell surface, where they were entangled with microvilli and eventually led to cell rupture. *S. agalactiae* is well known as an important pathogen frequently associated with bovine mastitis and a cause of huge economic losses in dairy herds [9]. In particular, *S. agalactiae* can colonize mammary epithelium and produce virulence factors that affect the physiological function of bMECs and can even result in cell death [10]. Some studies suggested that the pathopoiesis of *S. agalactiae* depended on the production of virulence factors such as capsular polysaccharides and surface proteins like Rib and hyaluronate lyase [11], and that the intracellular damage and induction of apoptosis in bMECs may be attributed to these virulence factors [12]. The results demonstrated that *S. agalactiae* possessed adhesion abilities and cytotoxic effects that could destroy cells in a relatively short time through apoptosis/necrosis.

The rapid inflammatory response stimulated by *S. agalactiae* has been confirmed in bMECs, and was characterized by release of proinflammatory cytokines, such as IL-1β, TNF-α and IL-8. It has been reported that IL-1β and TNF-α play critical roles in the host defense against infection and are responsible for early inflammatory responses [13]. In a mouse model of infectious mastitis, an increase in IL-1β and TNF-α levels was detected in the mammary glands after a 24-h intramammary challenge with *S. agalactiae* [14]. In agreement with this finding, we observed in bMECs incubated with *S. agalactiae* that IL-1β gene expression was significantly increased after 2 h. However, the level of TNF-α mRNA initially decreased after 1 h exposure to *S. agalactiae* and then increased again at 6 and 8 h. Although IL-6 is a pleiotropic cytokine that can be either pro- or anti-inflammatory [13], we found that exposure to *S. agalactiae* stimulated IL-6 mRNA expression in bMECs in a time-dependent manner. This was in line with a previous study that *S. agalactiae* stimulated a significantly greater release of IL-1β, IL-6, and TNF-α in mouse macrophages [15]. IL-8 is a critical chemotactic factor that can recruit neutrophils from the bloodstream to sites of infection [16]. In the present study, *S. agalactiae* induced a cellular inflammatory response in bMECs that inhibited the expression of IL-8 in the early stages of inflammation. Therefore, we concluded that the inflammation resulting from *S. agalactiae* infection could injure bMECs and reduce milk production.

In this study, proteomic analysis of bMECs infected by *S. agalactiae* was carried out by obtaining quantitative TMT measurements. Compared with control cells, we detected 325 up-regulated and 704 down-regulated proteins. Proteomic analysis revealed that these differentially expressed proteins included enzymes and proteins associated with various metabolic processes and cellular immunity. Our results also showed that some proteins were involved in metabolism of amino acids, sugars and purines, cell adhesion, cytoskeletal remodeling, protein processing and transporter functioning. Previous studies showed that the amount of milk produced by dairy cows was highly dependent on their body’s energy supply, and the physiological functioning of the mammary gland was closely related to the rate of carbohydrate metabolism [17]. In the present study, some differentially expressed proteins such as pyruvate kinase M2, fructose-1,6-diphosphatase 1/2, fructose diphosphate aldolase, and glyceraldehyde-3-phosphate dehydrogenase, participated in several carbohydrate metabolic pathways: galactose metabolism, glycolysis/glycogenesis, pyruvate metabolism, starch-sucrose metabolism and the pentose phosphate pathway. These proteins are important in the energy supply pathway, which can have a major impact on mammary gland function. Pyruvate kinase, which converts phosphoenolpyruvate to pyruvate is the key enzyme in the last step of glycolysis, and pyruvate kinase M2 is the key regulator of anaerobic glycolysis, which supplies energy for cells [18]. Likewise, fructose-1,6-diphosphatase, which catalyzes the conversion of fructose-1,6-diphosphate into fructose-6-phosphate in the glycolytic pathway [19] and glyceraldehyde-3-phosphate dehydrogenase which promotes ATP synthesis [20], were all significantly down-regulated after infection with *S. agalactiae* in the present study. Thus, the metabolism of bMECs was inhibited and ATP synthesis was reduced. This inhibition would have caused an energy and metabolic insufficiency that could affect cell proliferation, lactoprotein and lactose synthesis and milk production [21].

As a first-line of defense against pathogens invading the mammary gland, bMECs play a critical role in pathogen recognition and the innate immune response [22]. It has been reported that there are many pathogen recognition receptors (PRRs) on the cell membranes of bMECs, which can accurately identify the pathogen-associated molecular patterns (PAMPs) of many pathogens. This signal recognition triggers an immune response that promotes synthesis and release of various inflammatory factors, induces immune cell migration, and ultimately eliminates the pathogenic bacteria [23]. We found that among the down-regulated differential proteins, proteasome activator 1 [24], cathepsin B [25], cathepsin D [26] and cathepsin L2 [27] played important parts in the recognition of bacteria and in antigen presentation. In addition, claudin-3 (CLDN3) and claudin-4 (CLDN4) [28] are major structural molecules of the tight junctions that link epithelial cells, and together with junctional adhesion molecule A [29] are responsible for controlling leukocyte migration into tissues. These results were confirmed by the pathway enrichment results, which showed cell adhesion molecules (CAMs), glycolysis/gluconeogenesis, pentose phosphate pathway and purine metabolism.

We found that *S. agalactiae* may reduce the immune response in mammary tissue by blocking the pathogen recognition pathways and reducing leukocyte migration. Glutathione is an antioxidant tripeptide widely existing in cells, which has many important physiological functions, such as maintaining the stability of intracellular molecules and participating in amino acid transport [30]. Our results showed that the expression of glutathione *S*-transferase P1 (GSTP1), an enzyme that catalyzes the transfer of GSH to intracellular molecules, was down-regulated. Furthermore, it has been reported that GSTP1 is involved in glutathione metabolism and could reduce excessive ROS and maintain redox balance [31]. Other studies showed that GSTP1 inhibited cell apoptosis through a mitochondrial and MAPK-associated pathway [32]. In summary, these results may provide a better understanding of the mechanism of the injury caused by *S. agalactiae* and the apoptosis of bMECs, which can be tested by further research.

Among the top ten differentially expressed proteins, dimethylarginine dimethylaminohydrolase1 (DDAH1), peroxidase 6 (PRDX6), and epithelial cell adhesion molecule (EPCAM) deserve particular attention. DDAH1 is an enzyme that metabolizes methylated arginine to citrulline and methylamine. Dai et al. [33] stated that it may be related to tissue differentiation during breast development, and we speculate that it may be related to cell differentiation. Studies have shown that PRDX6 can prevent oxidative damage of mammary glands in mice [34]. The significant down-regulation of PRDX6 in this study may also mean that it is a target of *S. agalactiae*. EpCAM mediates cell-to-cell contact and recruits tight junction proteins to form an epithelial barrier against penetration by pathogenic bacteria and tissue invasion. Damage to the cells of the epithelial layer can lead to bacterial infection and cell death [35]. Hence, the decrease in EPCAM protein may reduce the integrity of bMECs in bovine mammary tissue allowing further tissue invasion by *S. agalactiae*. EPCAM also participates in apoptosis-related pathways, regulating the balance of the apoptotic promoter, Bax, and the anti-apoptotic molecule, Bcl-2. Gao et al. [36] showed that EpCAM could down-regulate the expression of Bcl-2 through an ERK1/2 signaling pathway, promote cell apoptosis, inhibit cell proliferation and cause cell cycle arrest. These effects are contrary to the results of our in vitro experiments and warrant further study. Histone H2B, non-histone chromosomal protein HMG-17 and transcription factor BTF3 are nuclear proteins that play important roles in cells. Further study on these proteins may discover new mechanisms in the process of *S. agalactiae* infection.

## Conclusion

Altogether, these results showed that incubation of bMECs with *S. agalactiae* induced an acute inflammatory response, promoted apoptosis and caused disruption of cell membranes and organelles. Our data suggest that *S. agalactiae* infection may affect cell proliferation and milk composition by inhibiting ATP synthesis. *S. agalactiae* may also avoid immune attack by inhibiting the pathogen recognition pathways and reducing the migration of immune cells. These results need to be further validated, but this study does suggest potential targets for blocking *S. agalactiae* from invading bovine mammary glands.
